# In Vitro Characterization of Twenty-One Antifungal Combinations against Echinocandin-Resistant and -Susceptible *Candida glabrata*

**DOI:** 10.3390/jof7020108

**Published:** 2021-02-02

**Authors:** Hazim O. Khalifa, Hidetaka Majima, Akira Watanabe, Katsuhiko Kamei

**Affiliations:** 1 Division of Clinical Research, Medical Mycology Research Centre, Chiba University, Chiba 260-8673, Japan; hazimkhalifa@chiba-u.jp (H.O.K.); majipulm@chiba-u.jp (H.M.); k.kamei@faculty.chiba-u.jp (K.K.); 2 Department of Pharmacology, Faculty of Veterinary Medicine, Kafrelsheikh University, Kafr El-Sheikh 33516, Egypt

**Keywords:** *C. glabrata*, echinocandins resistance, antifungal synergistic combinations, echinocandins, *FKS* mutations

## Abstract

This study was designed to analyze the interaction of 21 antifungal combinations consisting of seven major antifungal agents against 11 echinocandin- susceptible and six-resistant *C. glabrata* isolates. The combinations were divided into five major groups and were evaluated by checkerboard, disc diffusion, and time-killing assays. Synergy based on the fractional inhibitory concentration index of ≤0.50 was observed in 17.65–29.41% of the cases for caspofungin combinations with azoles or amphotericin B. Amphotericin B combination with azoles induced synergistic interaction in a range of 11.76–29.41%. Azole combinations and 5-flucytosine combinations with azoles or amphotericin B did not show synergistic interactions. None of the 21 combinations showed antagonistic interactions. Interestingly, 90% of the detected synergism was among the echinocandin-resistant isolates. Disk diffusion assays showed that the inhibition zones produced by antifungal combinations were equal to or greater than those produced by single drugs. The time-killing assay showed the synergistic action of caspofungin combination with fluconazole, voriconazole, and posaconazole, and the amphotericin B-5-flucytosine combination. Furthermore, for the first time, this assay confirmed the fungicidal activity of caspofungin-voriconazole and amphotericin B-5-flucytosine combinations. The combination interactions ranged from synergism to indifference and, most importantly, no antagonism was reported and most of the synergistic action was among echinocandin-resistant isolates.

## 1. Introduction

Fungal infections are gaining worldwide attention owing to their progressive increase, being responsible for than 1.6 million annual deaths worldwide, especially among immunocompromised patients and patients with severe immunosuppressive diseases [1]. Among fungal infections, special attention has been paid to *Candida* spp. infections, which were identified as the major cause of bloodstream infections in hospitalized patients [2]. *C. glabrata* is one of the most prevalent fungal pathogens worldwide and is considered the second most-common fungal cause of candidemia in the United States [3]. Infection with *C. glabrata* is challenging due to long hospitalization periods [4], high mortality rates [3], and the emergence of strains resistant to azoles, echinocandins, as well as multidrug-resistant strains [5,6].

Infections with *C. glabrata* are complicated as only few effective antifungal agents are available, due to its limited susceptibility to fluconazole [7]. For instance, since the discovery of the first antifungal agent (amphotericin B; 1950s) [8], only three antifungal classes (azoles, polyenes, and echinocandins) are currently indicated for the treatment of invasive candidiasis [2]. Echinocandins (caspofungin, micafungin, and anidulafungin) are used as first-line therapy for invasive candidiasis and act by inhibiting fungal cell wall synthesis through non-competitive inhibition of 1, 3-β-d-glucan synthase [3]. Since the introduction of the echinocandin drug caspofungin in 2001, significant progress in the treatment of *Candida* infections was achieved [3]. However, recent reports confirmed the increasing of echinocandin resistance in *C. glabrata* that has gained special attention [6,9,10].

Combination antifungal therapy might play a significant value in overcoming the emergence of echinocandin-resistant *C. glabrata*. This approach may increase the potency of fungal killing, mitigate the emergence of antifungal resistance, and extend the spectrum of activity, which subsequently shortens the antifungal therapy duration and reduces the mortality rates. Unfortunately, very little is known about the activity of antifungal combinations against *C. glabrata* [7,11,12]. To our knowledge, this is the first report evaluating a wide range of antifungal combinations against both echinocandin-resistant and -susceptible *C. glabrata* and the first to confirm that echinocandin-resistant isolates are more liable for antifungal synergism than echinocandin-susceptible isolates.

## 2. Materials and Methods

### 2.1. Fungal Strains

A total of 17 clinical echinocandin-resistant and -susceptible *C. glabrata* isolates were provided through the National Bio-Resource Project (NBRP) Japan (http://www.nbrp.jp/) [6,13]. Sixteen isolates were recovered from blood, and a single isolate was recovered from the nasal cavity. All isolates were previously confirmed and identified by the sequencing of the ITS1–5.8S–ITS2 region [14], and they were stored at −80 °C in 25% glycerol. Before the experiment, all of the isolates were subcultured on potato dextrose agar (Beckton Dickinson and Company, Sparks, MD, USA) to ensure viability and purity.

### 2.2. Antifungal Agents

In this study, seven antifungal agents representing the four major antifungal classes were tested alone or in combinations, forming 21 different combinations. The antifungals tested were caspofungin (CAS; Sigma–Aldrich, St. Louis, MO, USA), fluconazole (FLC; Sigma–Aldrich), itraconazole (ITC; Sigma–Aldrich), voriconazole (VRC; TCI America, Portland, OR, USA), posaconazole (POS; Sigma–Aldrich), amphotericin B (AMB; Sigma–Aldrich), and 5-flucytosine (5FC; Sigma–Aldrich). All antifungals were preserved in a dark environment in specific temperatures as recommended by the manufacturer.

### 2.3. Minimum Inhibitory Concentration (MIC) Determination

The MIC values for each antifungal alone were first determined by broth microdilution assay as recommended in CLSI document M27-Ed4 [15]. The breakpoints were evaluated according to CLSI document M60 [16], and *C. parapsilosis* ATCC 22019 and *C. krusei* ATTC 6258 were used as quality control strains. MIC values were interpreted visually after 24 h for all the isolates, with the exception of two isolates for which MIC was determined was after 48 h due to the absence of growth after 24 h [16]. Resistance to CAS and FLC was confirmed when the MIC values were >0.25 µg/mL and >32 µg/mL, respectively [16]. The MIC values were adopted according to epidemiological cut-off values for ITC (≥4), VRC (≥1), POS (≥4), AMB (≥4), and 5FC (≥1) [6,16].

### 2.4. Confirming Echinocandin Resistance

In *C. glabrata*, resistance to echinocandins is confirmed when an isolate is resistant to at least two echinocandins or when it harbors *FKS* gene hot spot (HS) mutations [10,17,18]. Therefore, all the isolates were checked genotypically to confirm the mutations in *FKS* genes HS regions including *FKS1* HS1, HS2, and HS3 and *FKS2* HS1 and HS2 as previously described using the primers listed in Table 1 [6]. Furthermore, the MIC value of micafungin (MFG) was previously tested as recommended in CLSI document M27-Ed4 by broth microdilution assay (data not shown) [6,13,15].

### 2.5. Checkerboard Assay

Antifungal combination activity was assessed by a checkerboard method derived from the standardized procedure established by the CLSI for broth microdilution antifungal susceptibility testing with a few modifications [15]. In brief, the evaluation was performed in RPMI 1640 medium buffered to pH 7.0 with 0.165 mol/L 3-(*N*-morpholino) propanesulfonic acid buffer (MOPS). Volumes of 25 µL of each drug at a concentration of four times the targeted final concentration were dispensed in the wells of 96-well microtiter plates (Violamo, Osaka, Japan). All antifungals were dissolved in DMSO as recommended by CLSI and the final DMSO concentration did not exceed that of the standard CLSI method. The final concentrations of the antifungal agents evaluated in this study ranged from 0.015 to 8.0 µg/mL for CAS, 0.5 to 32 µg/mL for FLC, 0.125 to 8 µg/mL for ITC, 0.03 to 2 µg/mL for VRC, 0.06 to 4 µg/mL for POS, 0.03 to 2 µg/mL for AMB, and 0.0009 to 0.06 µg/mL for 5FC. All concentrations were adopted according to the MIC results and within the CLSI-recommended concentrations [15], and, except for 5FC, the CLSI-recommended concentrations were very potent and induced complete inhibition of all isolates, so lower concentrations were evaluated [7]. Yeast inocula were adjusted to a 0.5 McFarland turbidity standard, and further diluted to two times final concentration. Yeast inoculum (50 µL) was added to every well to achieve final inoculum size of 0.5 × 10^3^ to 2.5 × 10^3^ CFU/mL. The plates were incubated at 35 °C for 24 h for all isolates, with the exception of two isolates, the plates were incubated for 48 h due to the absence of growth after 24 h [16]. Antifungal combination activities were evaluated on the basis of the fractional inhibitory concentration (FIC) index, and classified as synergistic, indifferent, or antagonistic [7,11,19]. The FIC index is expressed as ΣFIC = FICA + FICB = MICA^comb^/MICA^alone^ + MICB^comb^/MICB^alone^, where MICA^alone^ and MICB^alone^ are the MICs of every drug when acting alone, and MICA^comb^ and MICB^comb^ are the MICs of each antifungal in combination (in a single well). For all the antifungal combinations, the FICs were derived from IC50s [11]. The antifungal interaction was identified as synergistic if ΣFIC ≤ 0.50, indifferent if ΣFIC ranged from >0.50 to ≤4.0, and antagonistic if ΣFIC > 4.0 [7,11,19]. All antifungal combinations with synergistic action were repeated at least two independent times.

### 2.6. Disk Diffusion Assay

**Figure 1:**
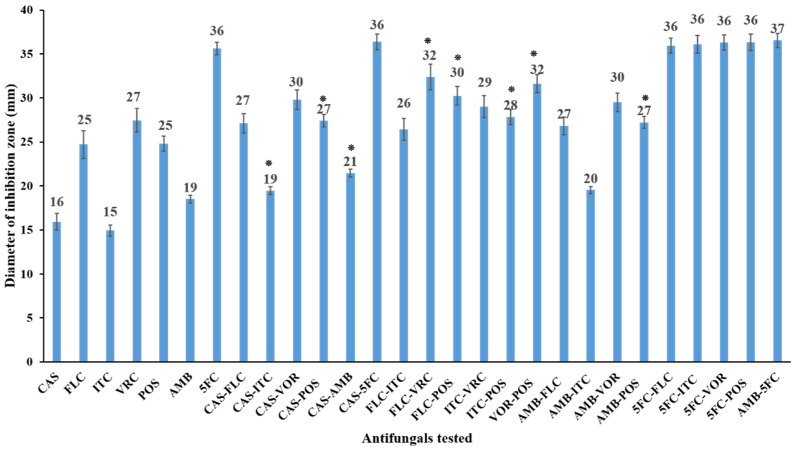
In vitro activities of CAS (4 µg/disk), FLC (25 µg/disk), ITC (4 µg/disk), VRC (1 µg/disk), POS (4 µg/disk), AMB (1 µg/disk), and 5FC (1 µg/disk), alone and in combinations against 17 *C. glabrata* isolates as determined by the disk diffusion assay. The results represent the mean of inhibition zone diameters ± standard deviation of the results from two independent experiments. The combinations showing statistical significance (*p* < 0.05) as compared to each corresponding single antifungal agent are marked with an asterisk (*).

Disk diffusion was performed for every drug individually or in combination as described in CLSI document M44, 3rd ed. [20]. Briefly, the yeast isolates were cultured on potato dextrose agar, and the yeast inoculum was adjusted to 0.5 McFarland standard. This was followed by plate streaking on Mueller–Hinton agar (Beckton Dickinson and Company, Sparks, MD, USA) supplemented with 0.5 µg/mL of methylene blue and 2% glucose. Sterilized disks (6-mm-diameter; Advantic, Toyo Roshi Kaisha, Ltd., Tokyo, Japan) were embedded with 10 µL of single drug or with drug combinations. The final concentrations for CAS, FLU, ITC, VOR, POS, AMB, and 5FC were 4, 25, 4, 1, 4, 1, and 1 µg/disk, respectively, which were corresponding to the doses described by CLSI-M44 and doses previously published by similar studies [12,20,21]. The inhibition zone diameters were measured after the plates were incubated for 24 h at 35 °C [20]. Before the experiment, the discs were evaluated with the CLSI recommended doses against the quality control strains, resistant, and susceptible *C. glabrata* strains and the generated inhibition zones were within the range documented by CLSI. The disk diffusion assay was performed in duplicate, and combined results of all 17 isolates were expressed as a mean diameter of inhibition zone for every drug alone or in combination (Figure 1).

### 2.7. Time-Killing Assay

The synergistic and/or the fungicidal activities of the antifungal combinations were determined by the time-killing assay as previously described [7,12,22]. Briefly, echinocandin-resistant *C. glabrata* IFM60089 was grown twice on potato dextrose agar plates, three to five colonies were suspended in 3–5 mL of sterile distilled water, and the turbidity was adjusted spectrophotometrically to a 0.5 McFarland. One ml of the 0.5 McFarland fungal suspension was added to 9 mL of RPMI 1640 medium buffered with MOPS with 20–50 µL of each drug alone or in combinations. To correlate with the disk diffusion assay, the same drug concentrations were used, with the exception of 5FC at 1 µg/mL, which induced a fungicidal effect within 6 h to the end of the experiment, so it was used at a concentration of 0.1 µg/mL. The test solutions were incubated in a shaker for 24 h at 35 °C. At 0, 2, 6, and 24 h following incubation, 100-µL aliquots were removed from each test solution and serially 10-fold diluted in sterilized distilled water. After dilution, 50-µL aliquots from the dilutions were plated in duplicate onto potato dextrose ager plates followed by incubation at 35 °C for 24–48 h for colony counting. The detection limit was 20 CFU/mL. In this assay, synergy was identified when the drugs in combination induced ≥100-fold increase in fungal killing compared with that obtained with the most active single antifungal. Antagonism was identified when drugs in combination induce ≥100-fold decrease in killing compared with that obtained with the most active single antifungal. The interaction was judged as indifferent when less than a 100-fold increase or decrease from the effect of the most active single antifungal was attained. Fungicidal activity of the combination was determined when the CFU/mL number was <99.9% compared with the initial fungal inoculum size [7,12,22]. The experiment was performed in duplicate, and the results expressed as log_10_ of the colonies count.

### 2.8. Statistical Analysis

Disc diffusion assay results were evaluated for statistical significance via *t*-test using Microsoft Excel 2016 (Microsoft Office 365, Microsoft Corp., Redmond, WA, USA). Every antifungal combination was compared against the corresponding individual antifungal agents and considered statistically significant when a *p*-value of <0.05 was obtained with both antifungal agents [7].

## 3. Results

### 3.1. Antifungal Susceptibility Profiling of the Tested Isolates

Echinocandin resistance was confirmed in six isolates that were resistant to at least two echinocandins and harbored *FKS* HS1 (five isolates with *FKS2* HS1 mutations and a single isolate with *FKS1* HS1 mutations), but not HS2 confirming its lower role for echinocandin resistance in *C. glabrata*. Additionally, there was no description of mutations in HS3 in clinical *Candida* spp. Furthermore, to evaluate the potential effect *FKS* redundant expression levels and/or echinocandin resistance level on the antifungal combination activity, four echinocandin-resistant isolates with *FKS2* S663P mutation from the same patient with different growth rates and different MIC values for echinocandins due to variations in the relative expression of redundant *FKS* genes as well as different MIC values for other antifungals were evaluated [6]. For azoles, only one isolate showed resistance to fluconazole and the rest were susceptible dose-dependently, while two isolates and a single isolate were noted to have MIC values higher than the epidemiological cut-off values for voriconazole and posaconazole, respectively (Table 2). All isolates had wildtype level sensitivity against itraconazole, amphotericin B, and 5-flucytosine (Table 2).

### 3.2. Checkerboard Assay

Antifungal combinations against the isolates were divided into five groups (Table 3). Generally, the MICs for all antifungal combinations were either lower than or equal to the MICs of the corresponding single antifungal and, most importantly, none of the previous combinations showed an antagonistic action (ΣFIC > 4.0). Median ΣFIC values for combinations including CAS were in the range of 0.56–1.14 for the six drug combinations, and synergistic action ranged from 17.65% to 29.41%, with the CAS-ITC combination being the most prevalent at 29.41% (Table 3). Azole combinations, 5FC combination with azoles, and 5FC combination with AMB did not induce any synergistic action, with median ΣFIC values of 0.79–0.90, 1.03–1.20, and 0.78, respectively (Table 3). Median ΣFIC values of AMB combination with azoles were in the range of 0.64–0.93, and synergistic actions ranged from 11.76% to 29.41%, with the AMB-POS combination being the most prevalent at 29.41% (Table 3). Interestingly, around 90% of the detected synergism was among the echinocandin-resistant isolates (Table 4 and Table 5). Furthermore, the four echinocandin-resistant isolates with *FKS2* S663P mutation from the same patient showed different patterns of interaction ranged from synergism to indifference (Table 4 and Table 5).

### 3.3. Disk Diffusion Assay

For further characterization of the effects of all 21 combinations, the disk diffusion assay was performed to test 17 clinical isolates. The results are shown in Figure 1. Overall, the inhibition zone diameters produced by antifungal combination therapies were either larger than or equal to those produced by the use of single antifungals. A significant increase *(p* < 0.05) of the inhibition zone diameter of the antifungal combination as compared with single antifungals used was achieved with CAS-ITC, CAS-POS, CAS-AMB, FLC-VRC, FLC-POS, ITC-POS, VRC-POS, and AMB-POS combinations. With the exception of the AMB-5FC combination, all 5FC combination zone diameters never exceeded the largest diameter obtained with 5FC alone. All other combinations showed indifferent effects, as the inhibition zone diameter was increased but was not statistically significant compared with single drugs (Figure 1).

### 3.4. Killing Assay

**Figure 2:**
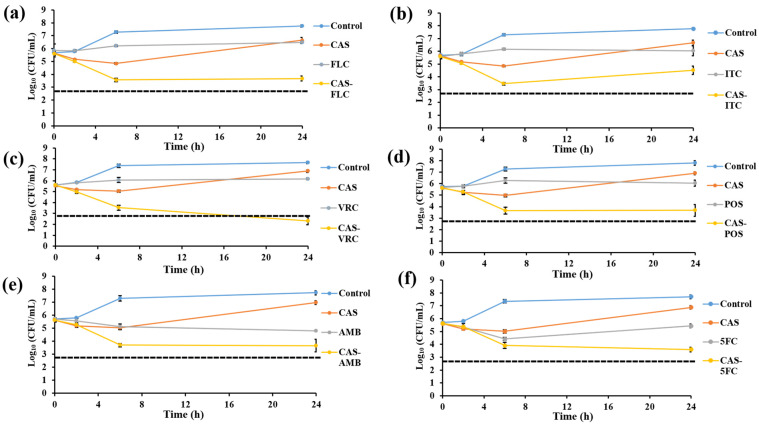
In vitro activities of CAS (4 µg/mL) in combination with FLC 25 µg/mL; (**a**), ITC (4 µg/mL; (**b**), VRC (1 µg/mL; (**c**), POS 4 µg/mL; (**d**), AMB 1 µg/mL; (**e**), and 5FC 0.1 µg/mL; (**f**) against *C. glabrata* IFM60089 as determined by the time-killing assay. The detection limit was 20 CFU/mL. Dashed lines represent a >99.9% growth reduction compared with the initial inoculum size for determination of fungicidal activity. CAS-FLC, CAS-VRC, and CAS-POS combinations showed synergetic activities, with only CAS-VRC combination showed fungicidal activity. Each data point represents the means ± standard deviation of results from two independent experiments.

**Figure 3:**
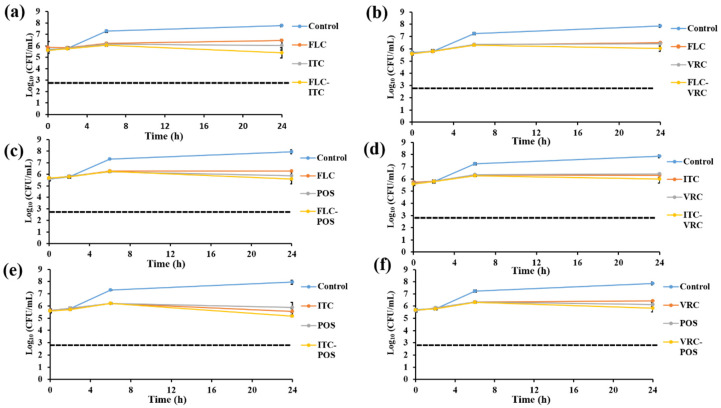
In vitro activities of azoles alone and in combinations including FLC-ITC 25–4 µg/mL (**a**), FLC-VRC 25–1 µg/mL; (**b**), FLC-POS 25–4 µg/mL; (**c**), ITC-VRC 4–1 µg/mL; (**d**), ITC-POS 4–4 µg/mL; (**e**), and VRC-POS 1–4 µg/mL; (**f**) against *C. glabrata* IFM60089 as determined by the time-killing assay. The detection limit was 20 CFU/mL. Dashed lines represent a >99.9% growth reduction compared with the initial inoculum size for determination of fungicidal activity. All azole combinations showed indifferent interactions. Each data point represents the means ± standard deviation of results from two independent experiments.

**Figure 4:**
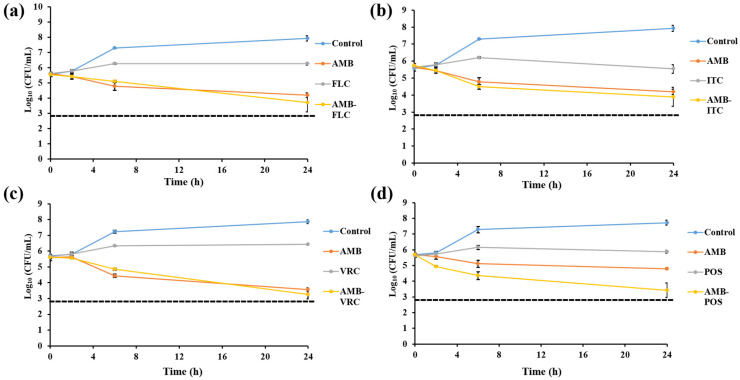
In vitro activities of AMB (1 µg/mL) alone and in combination with FLC 25 µg/mL; (**a**), ITC 4 µg/mL; (**b**), VRC 1 µg/mL; (**c**), and POS 4 µg/mL; (**d**) against *C. glabrata* IFM60089 as determined by the time-killing assay. The detection limit was 20 CFU/mL. Dashed lines represent a >99.9% growth reduction compared with the initial inoculum size for determination of fungicidal activity. All AMB combinations with azoles showed indifferent interactions. Each data point represents the means ± standard deviation of results from two independent experiments.

**Figure 5:**
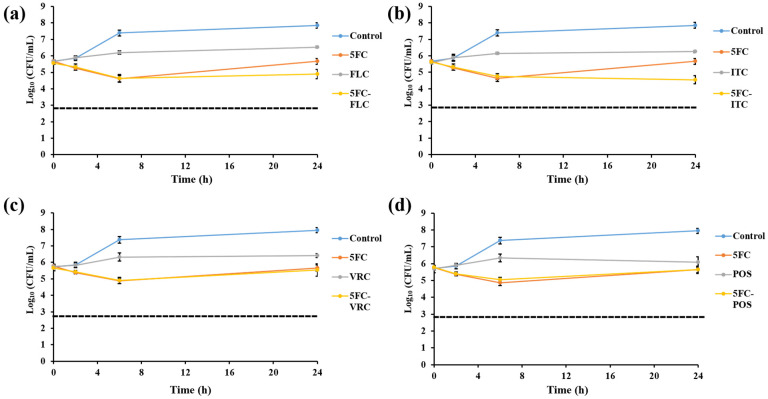
In vitro activities of 5FC alone (0.1 µg/mL) and in combination with FLC 25 µg/mL; (**a**), ITC 4 µg/mL; (**b**), VRC 1 µg/mL; (**c**), and POS 4 µg/mL; (**d**) against *C. glabrata* IFM60089 as determined by the time-killing assay. The detection limit was 20 CFU/mL. Dashed lines represent a >99.9% growth reduction compared with the initial inoculum size for determination of fungicidal activity. All 5FC combinations with azoles showed indifferent interactions. Each data point represents the means ± standard deviation of results from two independent experiments.

**Figure 6:**
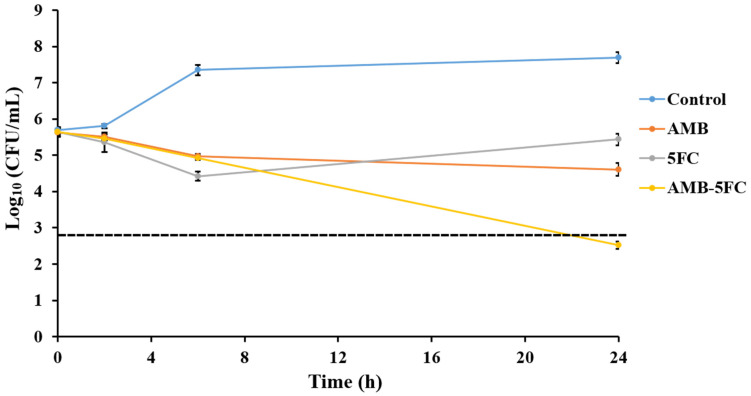
In vitro activities of AMB (1 µg/mL) and 5FC (0.1 µg/mL) alone and in combination against *C. glabrata* IFM60089 as determined by the time-killing assay. The detection limit was 20 CFU/mL. Dashed lines represent a >99.9% growth reduction compared with the initial inoculum size for determination of fungicidal activity. The AMB combination with 5FC showed synergistic and fungicidal actions. Each data point represents the means ± standard deviation of results from two independent experiments.

Time-killing assays were conducted with *C. glabrata* isolate IFM60089 as a representative of echinocandin-resistant isolates. This isolate was selected because it showed synergistic interaction for CAS-FLC, CAS-ITC, CAS-VRC, CAS-POS, and CAS-AMB combinations, while it showed indifference for all other combinations by the checkerboard assay. In general, all the combination therapies reduced the log_10_ CFU/mL to a greater degree than that achieved with single drugs (Figure 2, Figure 3, Figure 4, Figure 5 and Figure 6). For combinations including CAS, synergistic action was achieved with CAS-FLC, CAS-VRC, and CAS-POS combinations inducing 2.8, 3.84, and 2.36 log_10_ CFU/mL reduction more than that obtained with the most active single antifungals, respectively (Figure 2a,c,d). CAS-ITC, CAS-AMB, and CAS-5FC combinations achieved indifferent interactions yielding 1.53, 1.14, and 1.83 log_10_ CFU/mL reduction more than that obtained with the most active single drugs, respectively (Figure 2b,e,f). Interestingly, fungicidal effect was achieved with the CAS-VRC combination (Figure 2c). All azole combinations yielded indifferent interactions by achieving log_10_ CFU/mL reduction in a range of 0.29–0.64 more than that obtained with the most active single agents (Figure 3). Similarly, AMB or 5FC combinations with azoles induced indifferent interactions (Figure 4 and Figure 5). AMB combinations with azoles reduced log_10_ CFU/mL in a range of 0.30–1.37 than that obtained with AMB alone (Figure 4), while 5FC combinations with azoles reduced log_10_ CFU/mL in a range of 0.02–1.12 than that obtained with 5FC alone (most active antifungal) (Figure 5). Surprisingly, the AMB combination with 5FC induced synergistic and fungicidal actions by inducing 2.10 log_10_ CFU/mL reduction more than that obtained with the most active single antifungal (AMB; Figure 6). Antagonism interaction was never achieved among all the tested combinations.

## 4. Discussion

Antifungal combinations can be an attractive approach to overcome the emergence and spread of fungal resistance. This approach may have significant value for the treatment of *C. glabrata* for several reasons: first, this species is intrinsically resistant to fluconazole [6,7]; second, infections with *C. glabrata* have been confirmed to be associated with long hospitalization periods and high mortality rates as compared with another *Candida* spp. [3,4]; third, this species has the ability to acquire both azole and echinocandin resistance [5,6]; and, finally, the epidemiology of *C. glabrata* infections has recently been changed in different countries, and it has emerged as the second major cause of candidemia after *C. albicans* [3,6]. Despite these previous facts, evaluations of antifungal combination studies against *C. glabrata* are very rare [7,11,12].

Combinations of CAS with other antifungal agents, especially azoles and AMB, showed potent effects against the isolates, with 17.65% to 29.41% synergistic action by the checkerboard assay. For the first time, we document the synergistic action of the CAS-POS and CAS-AMB combinations and the fungicidal action of the CAS-VOR combination against *C. glabrata*. Furthermore, CAS-FLC, CAS-VRC, and CAS-POS synergistic actions were confirmed by the killing assay. More importantly, all synergistic actions with CAS were reported only against echinocandin-resistant isolates, which might have a value to overcome the rise of echinocandin-resistance in *C. glabrata*. To our knowledge, this is the first report to document that the antifungal resistance might affect the antifungal combination response in *C. glabrata*. Of note, CAS was used in this study as a representative for echinocandins, as the isolates showed low MFG MIC values, and difficult to get synergistic action with lower MFG concentrations. Azoles basically act by affecting ergosterol biosynthesis via inhibition of 14-alpha-demethylase [7], while AMB acts by the distribution of fungal cell membrane through pore formation after binding to ergosterol [8]. The difference in CAS, azole, and AMB mechanisms of actions might explain the synergistic action of their combinations [7,21]. Our killing assay results are in agreement with previous records showing the synergistic action of CAS-VOR and CAS-FLC and indifferent action of CAS-ITC against *C. glabrata* [12]. CAS-VRC, CAS-POS, and CAS-AMB combinations previously showed indifferent action against *C. glabrata* by a checkerboard assay [11], which is in agreement with our results as the majority of these combinations showed indifferent action.

Azole combinations seem to play a minor role in inducing a synergistic effect. With the exception of a significant increase of some azole combinations by the disk diffusion assay, no synergistic action was reported by the checkerboard assay or time-killing assay. Theoretically, antifungal combination synergism is expected when the evaluated antifungals have uniquely different mechanisms of action [7]. Our results are in agreement with previous speculations, as all the evaluated azoles possess the same mechanism of action. However, several experimental and clinical data elucidated that this speculation does not always hold true in antifungal combination therapy [7,23,24].

AMB is one of the major antifungals tested in combination with azoles to evaluate its enhanced activity when used in combinations against *C. albicans* [11,25,26,27,28]. On the other hand, information is scarce regarding its combination with azoles against *C. glabrata*. Our investigations showed its synergistic action with all tested azoles, ranging from 11.76–29.41% against the isolates by the checkerboard assay, and reduced log_10_ CFU/mL in the range of 0.30–1.37 by the time-killing assay. The synergistic combination of AMB-POS was previously reported against a single *C. glabrata* isolate [11], and AMB-VRC synergism was reported in 10% of tested *C. glabrata* isolates [7], which is in accordance with our findings. However, this is the first report elucidating the action of AMB-FLC and AMB-ITC against *C. glabrata*. Although AMB and FLC showed a promising result concerning the treatment of invasive candidiasis [28,29,30,31], the high rates of persistent candidemia by both drugs as well as the possibility of using higher doses of both drugs specially for non-*albicans* species of *Candida* are major concerns [28].

Due to the unique antifungal mechanism of action of 5FC by the inhibition of protein synthesis, its synergistic action with other antifungals has been evaluated [7,32,33,34]. Checkerboard investigations showed that synergistic action occurs rarely by 5FC combinations with all tested antifungal agents. These results might be attributable to the fact that all the tested isolates were highly susceptible to 5FC; the checkerboard assay was not able to detect any synergistic action due to its combinations. Furthermore, only the 5FC-AMB combination showed a fungicidal and synergistic action by the time-killing assay. As far as we know, this the first report to confirm the fungicidal and synergistic action of 5FC-AMB combination against *C. glabrata*. Clinically, 5FC-AMB combination at lower dosage with a prolonged course has a potent role for treatment of cryptococcal meningitis patients with fewer adverse effects [35]. Furthermore, time-killing assay results of 5FC combinations with other antifungal agents showed promising results with decreasing log_10_ CFU/mL in a range of 0.02–1.83 compared to that obtained when 5FC alone was tested. Classically, both in vitro and in vivo investigations confirmed that 5FC increased the activity of different azoles against *Cryptococcus neoformans* [32,33,34].

In this study, three different assays were evaluated in order to overcome the limitations of testing a single assay. For instance, although the checkerboard method is considered the standard method for evaluating the antimicrobial combinations, and previous reports confirmed the correlation between the laboratory evidence of ΣFIC synergy results with clinical evidence of optimal outcomes in insurgent yeast infections [11,36], dialectical results that can be obtained owing to variations in the standards used to evaluate the combination interactions, such as the evaluated concentrations, and the reading results and analysis methods [11]. Additionally, the calculated ΣFIC postulates that all antifungals interact with each other in a one-dimensional model (a linear model), allowing an all-or-none prospect, thus artificially creating original ΣFIC values [11,19,37]. The time-killing assay was developed to overcome checkerboard limitations, and it has the advantage of time-course evaluation of antifungal combination activity [11,38]. However, this assay is time-consuming and too labor-intensive, as well as limited tested drug concentrations, reading at a single time-point, and requirement for a fixed inoculum are problems with this assay [11,38]. The disk diffusion assay is a simple and time-efficient method for evaluating the antifungal combinations. However, synergistic, indifferent, or antagonistic interactions could not be distinctly identified by this assay [7,12].

Our investigations clearly demonstrated that the interaction of antifungal combinations is basically dependent on the evaluated assay, which is in agreement with previous reports [7,12]. Although the exact reason for the discrepancies is not clear, several hypotheses have been proposed [7,12,19,37]. The nature of the evaluated system, either static (checkerboard and disk diffusion assays) or dynamic (time-killing assay), might affect the pharmacodynamics of drug interaction [7]. In addition, the restricted evaluated dose ranges in disk diffusion and time-killing assays as compared to the widely evaluated dose ranges in the checkerboard assay might influence the results of the assays. Furthermore, our results showed, for the first time, that the synergistic action is more predominant among the echinocandin-resistant isolates and echinocandin resistance levels might affect the antifungals’ combination interaction. Therefore, other future studies are required to investigate the mechanism of action of antifungal combinations and reason(s) for response difference among the *Candida* isolates.

## 5. Conclusions

In conclusion, our investigations are very promising in terms of the fact that none of the twenty-one evaluated combinations showed antagonistic interactions by any evaluated assay. Although the majority of the combinations showed indifference effect, but importantly most of the detected synergism was among the echinocandin-resistant isolates. Several combinations showed synergistic interactions for the first time, such as CAS-POS, CAS-AMB, AMB-FLC, and AMB-ITC, against *C. glabrata*. Although time-killing assays were performed with only a single isolate, they provided encouraging results that confirmed the synergistic action of CAS-FLC, CAS-VRC, CAS-POS, and 5FC-AMB combinations, with CAS-VRC and 5FC-AMB combinations showing sustained fungicidal activity against an echinocandin-resistant isolate for the first time. However, in vivo and clinical studies are warranted to evaluate the safety and efficacy of the tested combinations before their clinical application.

## Figures and Tables

**Table 1:** Primers used for identification of *FKS* genes hot spots mutations.

Primer Name	Sequence	Target Gene/Purpose	PCR Product Sizes	Reference
FKS1-HS1,2,3-FW	GTCGCTACATTGCTATTTTTCTCAGTCATGCC	*CgFKS1* (HS1, HS2, HS3)/PCR and sequencing	2641 bp	[6]
FKS1-HS1,2,3-RV	CCATATAAATGGCAGAGCCTGCAAATCTGG	*CgFKS1* (HS1, HS2, HS3)/PCR and sequencing		
FKS1-HS1,3-RV1	GAGATAATGATAGCGTTCCAGACTTGGG	*CgFKS1* (HS1, HS3)/sequencing		[6]
FKS1-HS2-FW1	AAGATTGGTGCTGGTATGG	*CgFKS1* (HS2)/sequencing		[6]
FKS2-HS1,2-FW	CCATTAGGTGGTCTTTTCACCTCATATATGC	*CgFKS2* (HS1, HS2)/PCR and sequencing	2726 bp	[6]
FKS2-HS1,2-RV	GGATTAAATATGAATGGAGAGAACAGTAAAGCAG	*CgFKS2* (HS1, HS2)/PCR and sequencing		
FKS2-HS1-RV1	GCAAGTAAATGTTCTCTGTACATGG	*CgFKS2* (HS1)/sequencing		[6]
FKS2-HS2-FW2	TACTATGCGCATCCTGGTTTCCAT	*CgFKS2* (HS2)/sequencing		[6]

**Table 2:** Summary of antifungal susceptibility of 17 *Candida glabrata* isolates.

Drug ^1^	No. of Isolates at Each Determined MIC Value (µg/mL)														MIC Range (µg/mL)	GM
	≤0.008	0.015	0.03	0.06	0.12	0.25	0.5	1	2	4	8	16	32	≥64		
CAS							11	2			3			1	0.5–64	1.18
FLC									5	3	5	2	1	1	2–64	6.26
ITC							10	3	4						0.5–2	0.78
VRC				10	3	1	1	1			1				0.06–8	0.13
POS						1	10	4	1	1					0.25–4	0.69
AMB								7	10						1–2	1.5
5FC	2	13	2												0.008–0.03	0.015

^1^ CAS, caspofungin; FLC, fluconazole; ITC, itraconazole; VRC, voriconazole; POS, posaconazole; AMB, amphotericin B; 5FC, 5-flucytosine; GM, geometric mean.

**Table 3:** In vitro activities of twenty-one antifungal combinations divided into five groups by checkerboard assay against 17 clinical isolates *of Candida glabrata.*

Combinations ^1^	ΣFIC		% Isolates Showing the Following Interactions:		
	ΣFIC Range	Median ΣFIC	Synergism (Number/Total)	Indifference (Number/Total)	Antagonism (Number/Total)
1—Combinations including CAS					
CAS-FLC	0.38–1.5	0.76	23.53 (4/17)	76.47(13/17)	0 (0/17)
CAS-ITC	0.38–1	0.68	29.41 (5/17)	70.59 (12/17)	0 (0/17)
CAS-VRC	0.38–1.24	0.81	17.65 (3/17)	82.35 (14/17)	0 (0/17)
CAS-POS	0.28–0.75	0.61	17.65 (3/17)	82.35 (14/17)	0 (0/17)
CAS-AMB	0.25–0.75	0.56	23.53 (4/17)	76.47(13/17)	0 (0/17)
CAS-5FC	0.73–2	1.14	0 (0/17)	100 (17/17)	0 (0/17)
2—Azoles combinations					
FLC-ITC	0.56–1.13	0.90	0 (0/17)	100 (17/17)	0 (0/17)
FLC-VRC	0.61–1.25	0.82	0 (0/17)	100 (17/17)	0 (0/17)
FLC-POS	0.56–1.5	0.80	0 (0/17)	100 (17/17)	0 (0/17)
ITC-VRC	0.74–1.25	0.87	0 (0/17)	100 (17/17)	0 (0/17)
ITC-POS	0.56–1.5	0.79	0 (0/17)	100 (17/17)	0 (0/17)
VRC-POS	0.57–1.25	0.84	0 (0/17)	100 (17/17)	0 (0/17)
3—AMB with azoles					
AMB-FLC	0.25–1.02	0.93	11.76 (2/17)	88.24 (15/17)	0 (0/17)
AMB-ITC	0.19–1.02	0.84	11.76 (2/17)	88.24 (15/17)	0 (0/17)
AMB-VRC	0.09–1.03	0.80	11.76 (2/17)	88.24 (1517)	0 (0/17)
AMB-POS	0.31–1.24	0.64	29.41 (5/17)	70.59 (12/17)	0 (0/17)
4—5FC with azoles					
5FC-FLC	0.55–1.11	1.03	0 (0/17)	100 (17/17)	0 (0/17)
5FC-ITC	0.72–1.25	1.09	0 (0/17)	100 (17/17)	0 (0/17)
5FC-VRC	1.03–1.50	1.20	0 (0/17)	100 (17/17)	0 (0/17)
5FC-POS	1.05–1.12	1.07	0 (0/17)	100 (17/17)	0 (0/17)
5—AMB with 5FC	0.51–1.13	0.78	0 (0/17)	100 (17/17)	0 (0/17)

^1^ CAS, caspofungin; FLC, fluconazole; ITC, itraconazole; VRC, voriconazole; POS, posaconazole; AMB, amphotericin B; 5FC, 5-flucytosine.

**Table 4:** Complete characterization of the combinations with synergism.

No.	IFM	*FKS* Mutations	Echinocandin Resistance ^d^	Combinations ^a,b^								
				CAS-FLC	CAS-ITC	CAS-VRC	CAS-POS	CAS-AMB	AMB-FLC	AMB-ITC	AMB-VRC	AMB-POS
1	60089	*FKS1* S629P	R	+ (0.38)	+ (0.38)	+ (0.49)	+ (0.28)	+ (0.38)	-	-	-	-
2	61000		S	-	-	-	-	-	-	-	-	-
3	61017		S	-	-	-	-	-	-	-	-	-
4	61169		S	-	-	-	-	-	-	-	-	-
5	61186		S	-	-	-	-	-	-	-	-	-
6	61193		S	-	-	-	-	-	-	-	-	-
7	61743		S	-	-	-	-	-	-	-	-	-
8	61756		S	-	-	-	-	-	-	-	-	+ (0.38)
9	62339		S	-	-	-	-	-	-	-	-	+ (0.50)
10	64652		S	-	-	-	-	-	-	-	-	+ (0.50)
11	64679	*FKS2* S663P ^c^	R	+ (0.50)	+ (0.50)	-	-	-	-	-	-	-
12	64684	*FKS2* S663P ^c^	R	-	-	-	-	-	-	-	-	-
13	64686	*FKS2* S663P ^c^	R	+ (0.38)	+ (0.50)	+ (0.38)	+ (0.5)	+ (0.38)	+ (0.25)	+ (0.19)	+ (0.09)	+ (0.31)
14	64689	*FKS2* S663P ^c^	R	+ (0.50)	+ (0.38)	+ (0.38)	+ (0.38)	+ (0.25)	+ (0.31)	+ (0.38)	+ (0.50)	+ (0.50)
15	64903		S	-	-	-	-	-	-	-	-	-
16	64905		S	-	-	-	-	-	-	-	-	-
17	58273	*FKS2* F659del	R	-	+ (0.50)	-	-	+ (0.38)	-	-	-	-

^a^ CAS, caspofungin; FLC, fluconazole; ITC, itraconazole; VRC, voriconazole; POS, posaconazole; AMB, amphotericin B. ^b^ + Indicate synergism (FICI), - indicate indifference. ^c^ Four isolates with *FKS2* S663P from the same patient with different MIC values. ^d^ R indicate echinocandin resistant, S indicate echinocandin susceptible.

**Table 5:** Synergism percentage among echinocandin-resistant and -susceptible *C. glabrata* isolates.

Antifungal Combination Showed Synergistic Action ^1^	Echinocandin-Resistant Isolates (*n* = 6)	Echinocandin-Susceptible Isolates (*n* = 11)	Total (*n* = 17)
CAS-FLC	4/6 (66.66%)	0/11 (0%)	4/17 (23.53%)
CAS-ITC	5/6 (83.33%)	0/11 (0%)	5/17 (29.41%)
CAS-VRC	3/6 (50%)	0/11 (0%)	3/17 (17.65%)
CAS-POS	3/6 (50%)	0/11 (0%)	3/17 (17.65%)
CAS-AMB	4/6 (66.66%)	0/11 (0%)	4/17 (23.53%)
AMB-FLC	2/6 (33.33%)	0/11 (0%)	2/17 (11.76%)
AMB-ITC	2/6 (33.33%)	0/11 (0%)	2/17 (11.76%)
AMB-VRC	2/6 (33.33%)	0/11 (0%)	2/17 (11.76%)
AMB-POS	2/6 (33.33%)	3/11 (27.27%)	5/17 (29.41%)

^1^ CAS, caspofungin; FLC, fluconazole; ITC, itraconazole; VRC, voriconazole; POS, posaconazole; AMB, amphotericin B.

## Data Availability

The data are contained within the article.
